# Genetic Research and Plant Breeding

**DOI:** 10.3390/genes14010051

**Published:** 2022-12-23

**Authors:** Kwon-Kyoo Kang, Yong-Gu Cho

**Affiliations:** 1 Division of Horticultural Biotechnology, Hankyong National University, Anseong 17579, Republic of Korea; 2 Department of Crop Science, Chungbuk National University, Cheongju 28644, Republic of Korea

In the past 20 years, plant genetics and breeding research using molecular biology has been greatly improved via the functional analysis of genes, species identification and transformation techniques [1]. At the same time, innovations in molecular technology have spawned a new discipline called genomics [2]. The most epochal change among them is the development of next-generation sequencing (NGS) technology, which spurred research on the composition of regulatory regions and biological functions of many genes as the entire genome was deciphered with a focus on model plants. Functional genomics has become one of the most promising scientific areas in the characterization of gene (and protein) functions and interactions using vast genetics and various omics data [2]. In addition, there are various advanced tools and methodologies that could also enable the effective and efficient validation of gene functions when used in lab settings such as transcriptome, metabolomics, overexpression, knock-out, RNAi and gene editing, and when applying advanced computational methodologies such as machine learning and deep learning [2,3]. Farmers of the future can identify genetic differences between individual plants in a crop to make breeding-related inferences based on genomic information. The function of individual genes or DNA fragments can be inferred through links between genetic differences and emergence variations in various plants [1,4]. Genetic research also helps to characterize plants based on gene networks rather than individual genes. This allows plant breeding to understand and adapt to complex traits such as yield and tolerance to biotic and abiotic stresses. Therefore, to provide more information about gene function, scientists and farmers need to examine the genome, transcriptome, proteome and metabolites based on genome sequence data [1,5]. Based on these results, not only traditional breeding but also new and innovative breeding methods such as genome editing can be used to regenerate new plants for the future. Therefore, this Special Issue includes an agronomic characterization and gene function analysis using both molecular and computational biology in the laboratory to accelerate plant breeding programs. This topic includes a total of 25 articles: 22 research articles and 3 review articles. The articles in this topic focus on a variety of new tools and technologies to developing knowledge and resources for breeding yielding, climate-tolerant crop varieties.

## 1. Abiotic Stress Tolerance

An important factor in crop production is abiotic stress. Among abiotic stresses, heat and salt stress are regarded as the most critical factors affecting yield in most crops. this Special Issue includes two articles on abiotic stress tolerance research. In the first article, Jung et al. [6] found a resistance to heat stress in rice transformants overexpressing the OsOr or OsOr-R115H gene (Arg replaced by His at position 115 of the OsOr protein) and discussed their resistance mechanism. From these results, it was suggested that the OsOr-R115H gene can be used to develop heat-resistant rice varieties. In another article, Lim et al. [7] generated transgenic rice plants with single-copy/intergenic/homozygous overexpression *PsGAPDH* (*PsGAPDH*-OX) and investigated their responses to salinity stress. To elucidate the role of *PsGAPDH*-OX in the salt-stress tolerance of rice, an Illumina HiSeq 2000 platform was used to analyze transcriptome profiles of leaves under salt stress. The analysis results of sequencing data show that 1124 transcripts were differentially expressed. Using the list of differentially expressed genes (DEGs), functional enrichment analyses of DEGs, such as GO (Gene Ontology) terms and KEGG (Kyoto Encyclopedia of Genes and Genomes) pathways, were performed. KEGG pathway enrichment analysis revealed that unigenes exhibiting differential expressions were involved in starch and sucrose metabolism. Interestingly, *Trehalose-6-phosphate synthase* (*TPS*) genes, whose expression was enhanced by abiotic stress, showed a significant difference in *PsGAPDH*-OX. 

## 2. Biotic Stress Resistance

In addition, tolerance to biotic stress is one of the most important factors of crop production. This Special Issue contains three articles on biotic stress tolerance research. In the first article, Vanlay et al. [8] generated a total of 96 lines of interspecific and intraspecies hybrid rootstocks to improve tomato soil-borne disease resistance. The results show that the new hybrid combination improves horticultural traits and soil-borne disease resistance more than the commercial rootstock, Maxifort. Additionally, gray mold disease caused by *Botrytis* in onions (*Allium cepa* L.) during growth and storage negatively affects their yield and quality. In the second article, Lee et al. [9] searched for genes associated with gray mold resistance via a transcriptome analysis in onions. As a result, the resistant lines showed differences in expression patterns compared to the susceptible lines in jasmonate resistance 1 (JAR1), coronatine-insensitive protein 1 (COI 1), and transcription factor MYC2 (MYC2) genes. Additionally, *Sclerotinia stem rot* is one of the utmost important diseases in mustard, causing considerable losses in seed yield and oil quality. In the third article, Singh et al. [10] investigated the genetic pattern of *Sclerotinia* stem rot resistance in Indian mustard using six generations (P_1_, P_2_, F_1_, F_2_, BC_1_P_1_, and BC_1_P_2_), developed from the crossing of one resistant (RH 1222-28) and two susceptible genotypes (EC 766300 and EC 766123). Genetic analysis revealed that resistance was governed by duplicate epistasis. The comparative proteome analysis of resistant and susceptible genotypes indicated that peptidyl-prolyl cis-trans isomerase (A0A078IDN6 PPIase) showed high expression in resistant genotypes at the early infection stage, while its expression was delayed in susceptible genotypes.

## 3. Genetic Research on Gene Function

The genetic analysis of various genes of interest provides scientists with important information on their functions and gives useful insights to plant breeders for their breeding programs. Jeon et al. [11] performed the identification and characterization of *PTE-2*, a *Stowaway*-like MITE activated in transgenic Chinese cabbage lines. Transposable elements (TEs) such as PTE-2 make up a high proportion of the plant genome and contribute to genetic diversity and evolution, affecting genome structure or gene activity. Additionally, Kim et al. [12] identified and verified the genetic variation in transgenic cabbage (*Brassica rapa* ssp. pekinensis) by next-generation sequencing. The variation candidates that were expected to consistently occur in the transgenic lines were selected and validated. The single-nucleotide polymorphism (SNP) and insertion and deletion (InDel) candidates were identified using the resequencing data and validated by reverse transcription (RT)-PCR analysis. Xie et al. [13] analyzed of SI-related *BoGAPDH* family genes in *Brassica oleracea* L. and the response of *BoGAPC* to SI signals. A total of 16 members of the *BoGAPDH* family were identified in *B. oleracea*, which were conserved, distributed unevenly on chromosomes and had tandem repeat genes. Most of the genes were down-regulated during self-pollination, and the highest expression was found in stigmas and sepals. Additionally, different transcriptome data show that *BoGAPDH* genes were differentially expressed under stress, which was consistent with the results of qRT-PCR. The results of yeast two-hybrid and GST pull-down studies show that the SRK kinase domain interacts with the BoGAPC protein. The above results suggest that the *BoGAPDH* family of *B. oleracea* plays an important role in the process of plant stress resistance, and the *BoGAPC* gene may be involved in the process of self-incompatibility in *B. oleracea*, which may respond to an SI by directly encoding proteins interacting with SRK. In addition, plant tissue culture is an in vitro technique used to manipulate cells, tissues, or organs, and plays an important role in genetic transformation. However, plants cultured in vitro often exhibit unintended genetic and epigenetic variations. Park et al. [14] investigated DNA methylation level changes in transgenic Chinese cabbage plants (*B. rapa* ssp. *pekinensis*) and their effects on corresponding gene expression patterns. Differentially methylated regions (DMRs) of DNA between non-transgenic and transgenic lines were detected by bisulfite sequencing, and ten DMRs located in exonic regions were identified. The regions with methylation variations that were inherited and consistently maintained in the next-generation lines were selected and validated. Additionally, fuzzless mutants are ideal materials to decipher the regulatory network and mechanism underlying fuzz initiation and formation. Feng et al. [15] used two *Gossypium arboretum* accessions differing in fuzz characteristics to explore expression pattern differences and differentiate between genes involved in fuzz development using RNA sequencing. Weighted gene co-expression network analysis discerned the ME magenta module highly associated with a fuzz/fuzzless trait, which included a total of 50 hub genes differentially expressed between two materials. This module and hub genes identified will provide new insights on fiber and fuzz formation and be useful for the molecular design breeding of cotton genetic improvement. Additionally, decapitation is an essential agricultural practice and is a typical method for analyzing shoot branching. However, it is unclear exactly how decapitation controls branching. Dong et al. [16] reported that the decapitation of sunflower plants led to the development of lateral buds, accompanied by a decrease in indole-3-acetic acid (IAA) and abscisic acid (ABA) levels and an increase in cytokinin (CK) levels. Additionally, 82 members of the HabZIP family were discovered and categorized into nine groups using phylogenetic and conservative domain analyses. Based on tissue-specific expression and expression analysis following decapitation derived from the transcriptome, several HabZIP members may be involved in controlling decapitation-induced bud outgrowth. Therefore, it is hypothesized that the dynamic variations in hormone levels, in conjunction with particular HabZIP genes, led to the development of axillary buds in sunflowers following decapitation. In addition, the LBD (lateral organ boundaries domain) family are a new group of plant-specific genes, which encode a class of transcription factors containing conserved lateral organization boundary (LOB) domains, and play an important role in regulating the adaxial–abaxial polarity of plant leaves. Lin et al. [17] isolated the *BcAS2* gene from the pak choi cultivar “NHCC001”, and analyzed its expression pattern. The results show that the *BcAS2* encoded a protein made up of 202 amino acid residues located in the nucleus and cytomembrane. The yeast two-hybrid system (Y2H) assay indicates that *BcAS2* interacts with *BcAS1-1* and *BcAS1-2* (the homologous genes of *AS1* gene in pak choi). In the transgenic *Arabidopsis thaliana* that overexpresses the *BcAS2* gene, it presented an abnormal phenotype with a curly shape. Taken together, our findings not only validate the function of *BcAS2* in leaf development in *A. thaliana*, but also contribute to unravelling the molecular regulatory mechanism of *BcAS2*, which fulfills a special role by forming complexes with *BcAS1-1/2* in the establishment of the adaxial–abaxial polarity of the lateral organs in pak choi. Additionally, tetraploid plants often have altered rates of vegetative growth relative to their diploid progenitors. However, the molecular basis for altered growth rates remains a mystery. Xu et al. [18] reports microRNA (miRNA) and gene expression differences in *Populus* tetraploids and counterpart diploids using RNA and miRNA sequencing. The results show that there was no significant difference between young leaves in the expression of vegetative growth-related miRNAs. However, as the leaves aged, the expression of auxin- and gibberellin-related miRNAs was significantly upregulated, while the expression of senescence-related miRNAs was significantly downregulated. As a result, the chloroplast degradation of tetraploid leaves was accelerated, the photosynthetic rate decreased, and the synthesis and decomposition ability of carbohydrate decreased. Additionally, root network structure plays a crucial role in growth and development processes in rice. Longer, more branched root structures help plants to assimilate water and nutrition from soil, support robust plant growth, and improve resilience to stresses, such as disease. Understanding the molecular basis of root development through the screening of root-related traits in rice germplasms is critical to future rice breeding programs. Zhang et al. [19] used a small germplasm collection of 137 rice varieties chosen from the Korean rice core set (KRICE_CORE) to identify loci linked to root development.

## 4. Molecular Breeding Based on SNP Markers

The development of an efficient, robust and high-throughput SNP genotyping platform is greatly significant as it plays a pivotal role in crop genetics and breeding. Lee et al. [20] studied the development and applications of a target capture-sequencing SNP-genotyping platform in rice. This platform was used in the diversity analysis of 50 rice varieties. The 2341 of 2565 SNP markers produced useful polymorphic genotype data, enabling the production of a phylogenetic tree of the 50 varieties. The platform was used for the QTL mapping of PHS (preharvest sprouting) resistance in an F_8_ recombinant inbred line population derived from the cross Odae × Joun. This strategy is a powerful tool for breeding and rice genetics and facilitates gene mapping and QTL studies, germplasm diversity analysis, and marker-assisted selection. Additionally, NGS (next-generation sequencing technologies) have enabled the discovery of a lot of sequence variations among closely related crop varieties. Ji et al. [21] used a set of resequencing data from 24 Korean japonica rice varieties in a temperate region and discovered 954,233 sequence variations, including 791,121 SNPs (single nucleotide polymorphisms) and 163,112 InDels (insertions/deletions). The SNP variants were classified into four groups according to their impact: high, moderate, low, and modifier. These groups contain 3524 (0.4%), 27,656 (2.9%), 24,875 (2.6%), and 898,178 (94.1%) variants, respectively. These results are useful for developing new markers for MAS (marker-assisted selection), selecting candidate genes in map-based cloning, and producing efficient high-throughput genome-wide genotyping systems for Korean temperate *japonica* rice varieties. In addition, Kim et al. [22] measured the thickness of seed coat and aleurone layers using a set of 294 rice varieties. They also found candidate genes for selecting the thickness of seed coat and aleurone layers by performing GWAS analyses using whole-genome resequencing data. Two primer pairs that can be used as HRM (high-resolution melting) markers were developed using HRM markers and the genotyping of BC_2_F_2_ individuals derived from a cross between Samgwang and Seolgaeng. These authors yielded reliable results to finally develop HRM markers for selecting seed coat and aleurone layer thickness. Additionally, molecular breeding that involves marker-assisted selection (MAS) addresses the limitations of conventional breeding and allows the pyramiding of multiple useful genes into a single cultivar. The utilization of DNA markers in a marker-assisted backcrossing (MABc) program significantly increases selection efficiency. Kim et al. [22] attempted to develop new breeding lines with a thinner seed coat and aleurone layer to provide a high eating quality with softer chewing characteristics and processability in rice grain. Line selection according to genotype of KASP markers was successful in the BC_1_F_1_ and BC_2_F_1_ generations, with the recurrent parent genome recovery ratio ranging from 91.22% to 98.65%. In BC_2_F_1_ seeds of the selected lines, the thickness of the aleurone layer was found to range from 13.82 to 21.67 μm, which is much thinner than the 30.91 μm of the wild type, suggesting that selection by MABc could be used as an additional breeding material for the development of highly processed rice varieties. Additionally, Scariolo et al. [23] performed an in-depth characterization of 15 varietal clones belonging to two distinct *Lavandula* species by means of a restriction-site-associated DNA sequencing (RAD-Seq). These authors demonstrated that this technology screens single-nucleotide variants that can access the genetic identity of individual accessions, reconstruct genetic relationships among related breeding lines, group them into genetically distinguishable main subclusters, and assign their molecular lineages to distinct ancestors. Overall, the results highlight the presence of pure ancestries and interspecific hybrids for the analyzed *Lavandula* species and demonstrate that RAD-Seq analysis is very informative and highly reliable for characterizing *Lavandula* clones and managing plant variety protection. The review article by Yu et al. [24] addresses how breeders persistently supply farmers with the best varieties in order to exceed consumer demand through resource-intensive plant-breeding processes. In order to motivate continuous innovation in variety development, a system needs to provide incentives for plant breeders to develop superior varieties, for example, exclusive ownership to produce and market those varieties. The most common system is the acquisition of intellectual property protection through plant variety protection, also known as breeder’s right. Most countries have adopted the system established by the International Union for the Protection of New Varieties of Plants (UPOV). To be granted plant variety protection, the variety should prove to be unique by meeting three requirements: distinctness, uniformity, and stability.

## 5. Genetic Research on Plant Development

An important review on the interspecific hybridization of transgenic *B. napus* and *B. rapa* was compiled by Sohn et al. [25] The authors also found that intraspecific hybridization among the *Brassica* species is very common, especially between *B. napus* and *B. rapa*. In general, interspecific hybrids cause numerous genetic and phenotypic changes in parental lines. Consequently, their fitness and reproductive ability are also very diverse. Additionally, the plant leaf, the main organ of photosynthesis, is an important regulator of growth. Zhang et al. [26] investigated leaf length, width, thickness, area, leaf mass per area (LMA), and cell size from two genotypes and profiled the transcriptome-wide gene expression patterns through RNA sequencing. The results show that the leaf area of Pd was significantly larger than that of Ps, but the epidermal cell area was significantly smaller than that of Ps. The difference in leaf size was caused by cell numbers. A transcriptome analysis also revealed that genes related to chromosome replication and DNA repair were highly expressed in Pd, while genes such as the *EXPANSIN* (*EXPA*) family, which promoted cell expansion, were highly expressed in Ps. Therefore, these data provide a valuable resource for understanding leaf development in the *Populus* genus. Additionally, seed coat color is a crucial agronomic trait in sesame (*Sesamum indicum* L.) since it is strongly linked to seed oil, proteins, and lignans contents and influences consumer preferences. In East Asia, black sesame seeds are used for the treatment and prevention of various diseases. However, in sesame, little is known about the establishment of the seed coat color, and only one gene has been reported to control black pigmentation. Wang et al. [27] provide an overview of developing seeds transcriptome of two varieties of sesame “Zhongfengzhi No.1” (white seed) and “Zhongzhi No.33” (black seed) and shed light on genes involved in black seed formation. Litvinov et al. [28] found that the development of new, more productive varieties of agricultural crops is becoming an increasingly difficult task. Modern approaches to the identification of beneficial alleles and their use in elite cultivars, such as quantitative trait loci (QTL) mapping and marker-assisted selection (MAS), are effective but insufficient for keeping pace with the improvement of wheat or other crops. Metabolomics is a powerful but underutilized approach that can assist with crop breeding. The article summarizes basic methodological information, and the current strategies of applications of metabolomics related to crop breeding are explored using recent examples.

## 6. Summary

In summary, this Special Issue comprises a diverse collection of both original research and review articles. These articles generated a large amount of genetic and genomic resources in different crops, including rice, sesame, Chinese cabbage, *B. oleracea,* sunflower, tomato, onion, Indian mustard and poplar, etc. The articles in this topic reported the application of many modern genomic tools for crop improvement, such as transgenic approach, GWAS, high-density marker platforms, genotype by sequencing, marker-assisted and genomic selection, single-molecule long-read sequencing technology, transcriptome analyses and candidate gene analyses, etc. We believe that the resources and knowledge generated through articles published in this Special Issue will help to stimulate crop improvements, thus playing an important role in increasing crop production for future food security.
